# Role of calcifying nanoparticles in the development of testicular microlithiasis in vivo

**DOI:** 10.1186/s12894-017-0289-0

**Published:** 2017-10-30

**Authors:** Xia-cong Lin, Xiang Gao, Gen-sheng Lu, Bo Song, Qing-hua Zhang

**Affiliations:** 1 Department of Urology, the 175th Hospital of PLA (Dongnan Affiliated Hospital of Xiamen University), Zhangzhou, Fujian 363000 People’s Republic of China; 2 Department of Obstetrics and Gynecology, Daping Hospital, Third Military Medical University, Chongqing, 400038 People’s Republic of China; 3 Urological Research Institute of PLA, Southwest hospital, Third Military Medical University, Chongqing, 400038 People’s Republic of China

**Keywords:** Calcification, Calcifying nanoparticles, Inflammation, Testicular microlithiasis, seminiferous tubules

## Abstract

**Background:**

Calcifying nanoparticles (NPs) have been proven to be associated with a variety of pathological calcification and previously detected in semen samples from patients with testicular microlithiasis (TM). The present study was designed to test the hypothesis if human-derived NPs could invade the seminiferous tubules and induce TM phenotype.

**Methods:**

The animals were divided into three groups. Normal saline (0.2 mL) was injected into the proximal right ductus deferens in group A as a control group. The experimental groups, B and C received *Escherichia coli* (10^6^ cfu/mL, 0.2 mL) and human-derived NPs suspension (0.2 mL), respectively. Rats were euthanized in 2 batches at 2 and 4 weeks. Testicular pathology, ultrastructure and inflammatory mediators were assessed.

**Results:**

Chronic inflammatory changes were observed at 2 weeks in both groups B and C. Moreover, the innermost layer of sperm cells were structurally impaired and a zone of concentrically layered collagen fibers around the human NPs body was formed in the lumen of the seminiferous tubule in group C only, in which TM phenotype of remarkable calcification surrounded by cellular debris within the seminiferous tubules was built at 4 weeks.

**Conclusions:**

The results obtained from our study suggested a potential pathogenic effect of NPs in the development of calcification within the seminiferous tubules, which should be addressed in the future studies.

## Background

Testicular microlithiasis (TM) is an uncommon condition of unknown etiology with multiple tiny calcifications present within the seminiferous tubules [1–3], which is associated with both malignant and benign conditions such as testicular neoplasms [4, 5]. So far no valid clinical or research approaches used to investigate the activity of TM in malignant conditions and infertility are available because of its unknown etiology. From clinical and laboratory studies, the existing evidence has confirmed that TM is formed by the intratubular deposits consisting of calcified central cores surrounded by multiple concentric layers of cellular debris, glycoprotein, and collagen within the seminiferous tubules [6]. However, possible pathophysiological mechanisms leading to the formation of calcified central cores of TM are not yet fully understood. Recently, nano-sized bacteria-like organisms (nanobacteria) have been proven to be involved in the process of pathological calcifications and previously detected in semen samples from patients with testicular microlithiasis (TM) [6–10].

The terminology ‘nanobacteria’ referring to nano-sized bacteria-like organisms were first discovered in a cell culture and named by Kajander and Cifcioglu [7]. They were self-replicating, 0.1–0.5 μm in size, and had the capacity of forming calcium phosphate minerals under subsaturation levels of calcium and/or phosphate [8, 9]. Nanobacteria, as an emerging cause of pathological calcification, were therefore also called calcifying nanoparticles (NPs). Although there is insufficient proof that calcifying nanoparticles are living organisms, a great deal of evidence from previous studies has established a possible relationship between nanoparticles and pathological calcifications, such as aortic valve calcification, prostatic calculi, renal tubular calcification, and dental pulp stone [6–10]. This study was to test the hypothesis if human-derived NPs could invade the seminiferous tubules and induce TM phenotype.

## Methods

### Generation and preparation of human NPs

Referring to our previous research methods [10], nine patients with TM (multiple foci <3 mm in diameter in testicular parenchyma with sonography) were recruited and gave informed consent to participate in the investigation, which was approved by the local Institutional Review Board of our department. According to the culture techniques described by Kajander and Ciftçioğlu [7], after semen and urine samples were pretreated with oscillation, diluted, filtrated (using pinhole filter, 0.45 and 0.22 μm, Millex; Millipore Carrigtwohill, Cork, Ireland), and centrifugated, the samples were routinely cultured in flasks containing serum-free RPMI-1640 (GIBCO, Invitrogen, Carlsbad, CA, USA) medium at 37 °C (pH 7.4) in 5% CO2/95% air. After 5 weeks of inoculation, cultures were harvested by 30-min centrifugation at 20,000×g and resuspended in 10 ml PBS (pH 7.2) to prepare human NPs suspension under the same ionic condition in normal saline (1 McFarland U) for further experiments.

### Rats infections with human NPs

All animal experiments were approved by the Institutional Animal Care and Use Committee. Sixty male Sprague Dawley rats (6 months old, 300–350 g) were divided into three groups: group A (control) with normal saline (0.2 mL) injected into the proximal right ductus deferens; group B receiving *E. coli* (10^6^ cfu/mL, 0.2 mL); group C receiving human-derived NPs suspension (0.2 mL).

### Sample collection

All rats were raised with the same conditions and were euthanized by asphyxiation with carbon dioxide (CO_2_) followed by cervical dislocation in 2 batches(ten rats in each)at 2 and 4 weeks, respectively. By aseptic technique the testis and epididymis were harvested before the epididymis was completely removed.

### Histological staining

Testis was fixed in 10% buffered formaldehyde for 24-48 h, and then embedded in paraffin. The paraffin blocks were cut into 3-μm thick pieces and then stained with hematoxylin and eosin.

### Testis ultrastructural study

Referring to our previous research methods [11], for transmission electron microscopy (TEM), fresh testis tissue were cut into 1–2 μm pieces, fixed overnight in 2.5% glutaraldehyde, embedded on a membrane coated copper screen, and stained with 3% phosphotungstic acid for 1–2 min before being viewed on a Tecnai 10 transmission electron microscope (Philips, Eindhoven, The Netherlands) with an 80 kV working voltage.

For scanning electron microscopy (SEM), fresh testis tissues were cut into small blocks before being analyzed. Briefly, tissues were fixed for 1 h with 2.5% glutaraldehyde and dehydrated in an ethanol gradient, followed by cryodesiccation and metal plating. Samples were observed under a KYKY-EM3200 SEM (KYKY Technology Development, Beijing, People’s Republic of China) using an accelerating voltage of 30 kV.

### Statistical analysis

Statistical analysis was performed with the unpaired student *t* test or with a one-way analysis of variance with the Tukey post hoc test. Data were processed using the SPSS statistical software (version 16.0; SPSS, Inc., Chicago, IL, USA). Significance was accepted at a *P* value of less than 0.05.

## Results

**Fig. 1:**
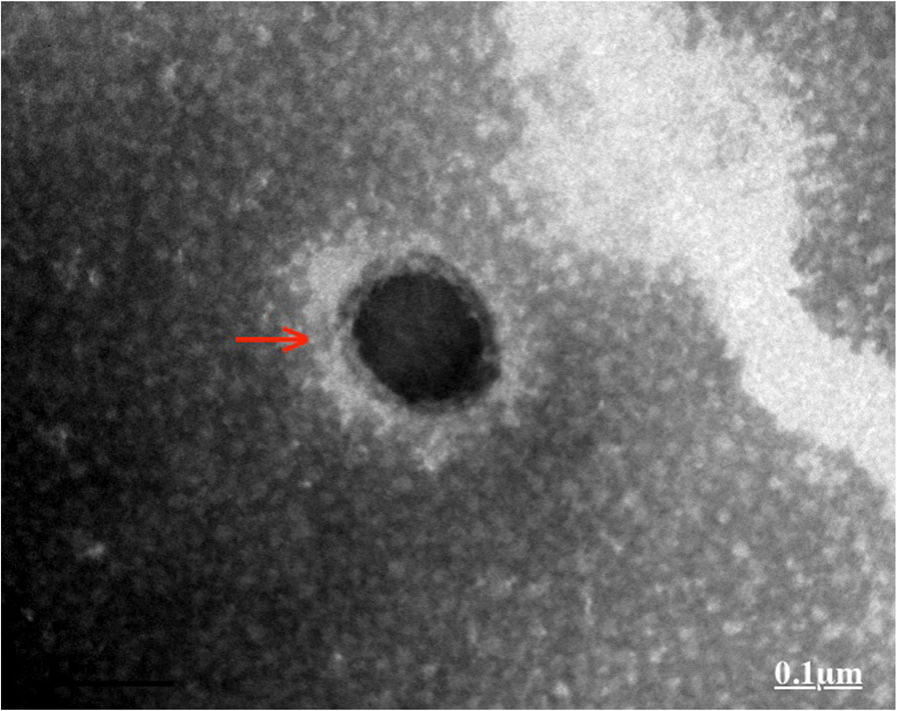
TEM showed that the cultured microbes were spheroid and about 80–200 nm in size with an apparently thick cellular wall (red arrow), which are homoplastic features to those of human NPs described in previous studies

Human NPs, existing as white granular sediments in cultures, were isolated from semen samples of TM patients. Under TEM, the NPs displayed as crystalline spheroids with approximately 80–200 nm in size (Fig. 1).

### Histopathological findings

**Fig. 2:**
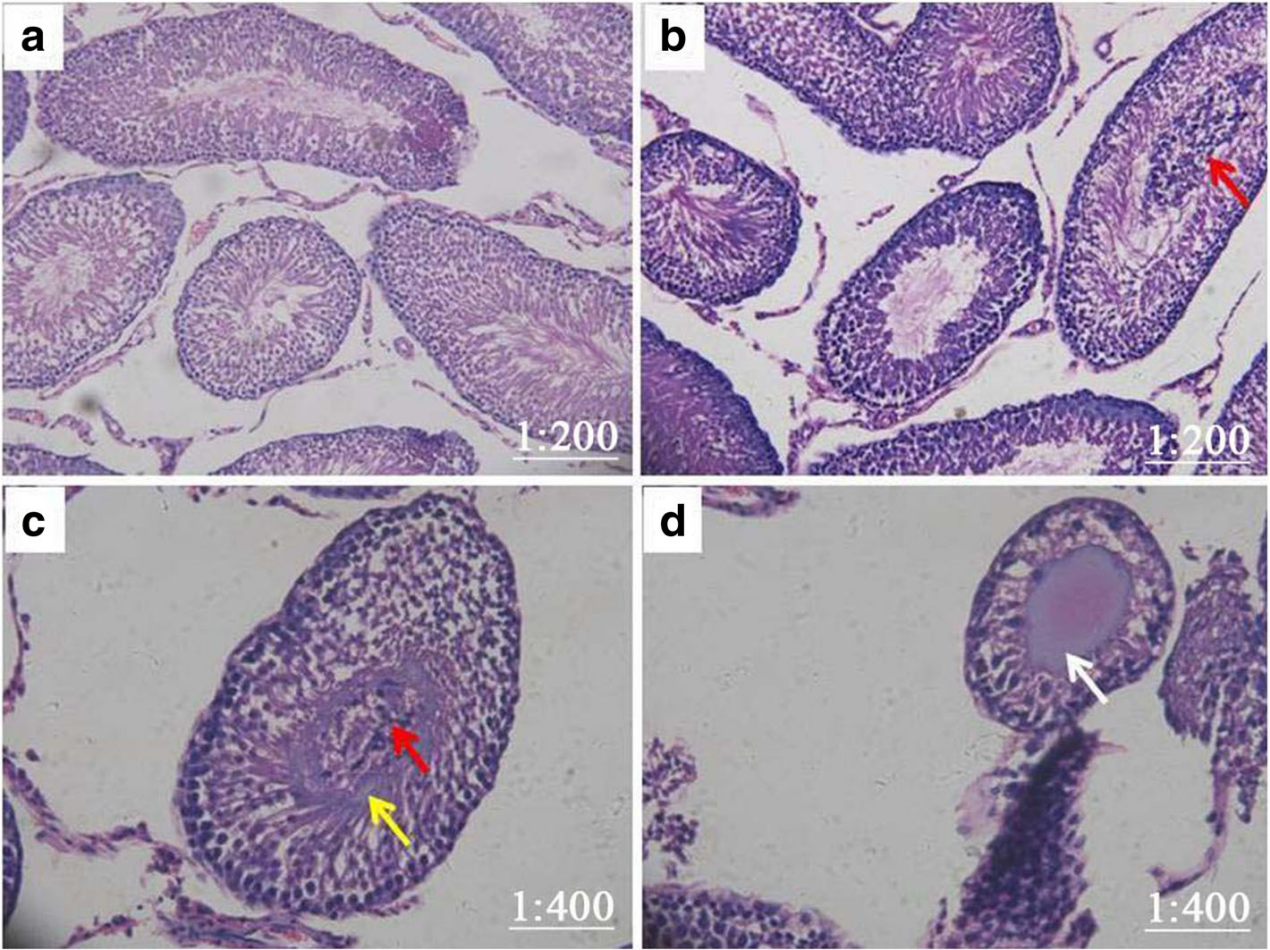
Effect of *E. coli* and human NPs on testis histopathology. **a** The testes from control mice showed normal morphology and spermatogenesis. H&E, reduced from ×200. **b** and **c** At 2 weeks, significantly leukocytic infiltration changes in the innermost layer of sperm cells were noted in groups B and C (Fig. 2b and c; red arrows). In addition, the innermost layer of sperm cells were structurally impaired and a zone of concentrically layered collagen fibers around the human NPs body was formed in the lumen of the seminiferous tubule in group C (Fig. 2c; yellow arrow). H&E, reduced from ×400. **d** At 4 weeks after infusion, seminiferous tubule was atrophic and a well-recognised form of intratesticular calcification surrounded by cellular debris within the seminiferous tubules was observed in group C only (white arrow), indicating a model of TM phenotype successfully reproduced. H&E, reduced from ×400

The control group revealed normal histological features, which were characterized by regularly-organized distribution of cells in the seminiferous epithelium, including the outermost layer of spermatogonia and Sertoli cells, the middle layer of spermatocytes, and the innermost layer of sperm cells (Fig. 2a). At 2 weeks, significant leukocytic infiltration changes in the innermost layer of sperm cells were observed in groups B and C (Fig. 2b and c). In addition, the innermost layer of sperm cells were structurally impaired and a zone of concentrically layered collagen fibers around the human NPs body was formed in the lumen of the seminiferous tubule in group C (Fig. 2c). At 4 weeks, atrophy of seminiferous tubule was observed and a well-recognized form of intratesticular calcification surrounded by cellular debris within the seminiferous tubules following inflammatory disease resulting from NP infusion was formed in group C only (Fig. 2d), which indicated the establishment of a successfully reproduced model exhibiting the TM phenotype.

### Ultrastructural observations

**Fig. 3:**
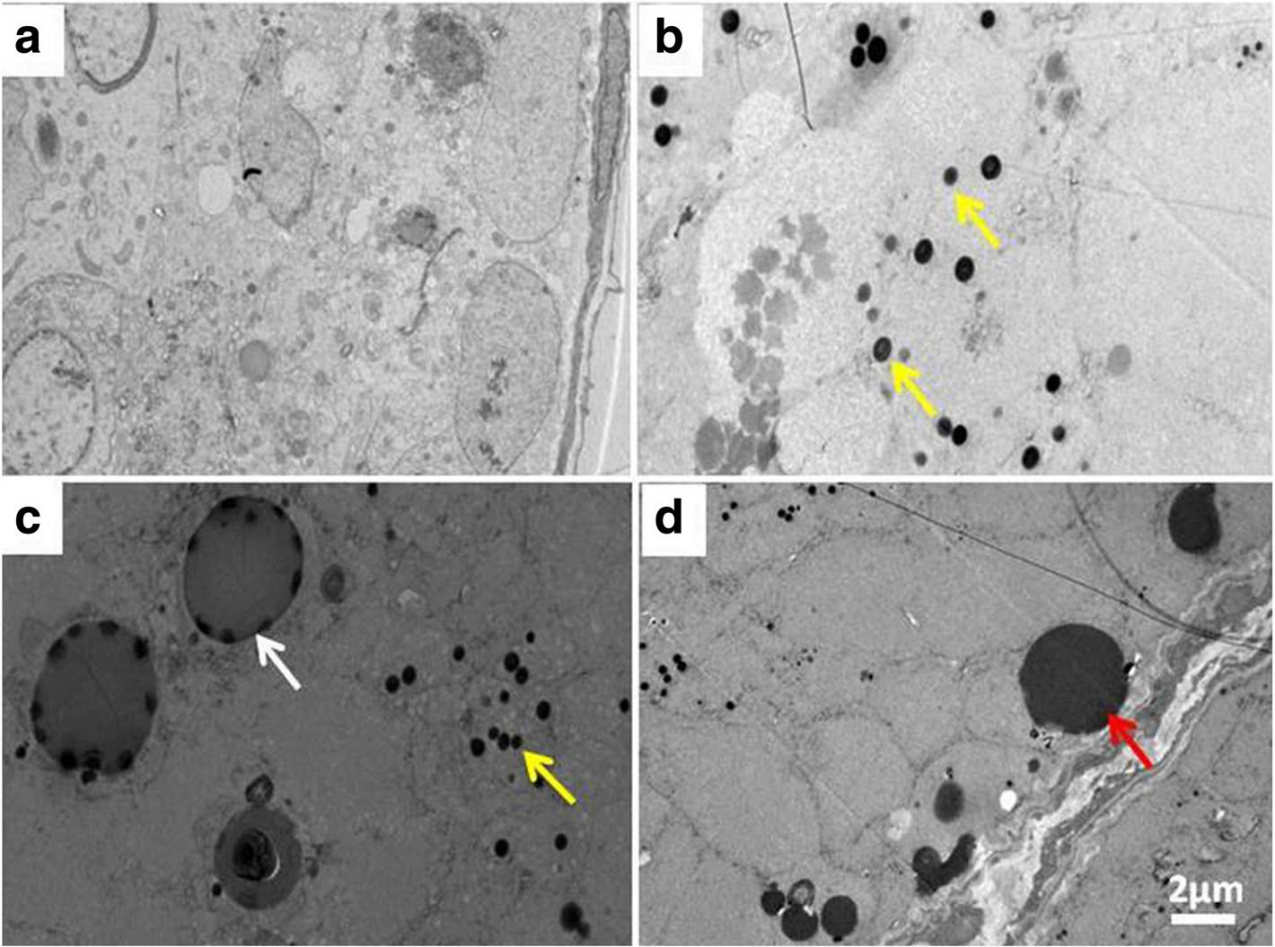
Testis ultrastructural observations by TEM. **a** Normal cell population typically localized in the seminiferous tubules and no morphological changes were observed in the testis of control mice. **b** and **c** At 2 weeks, many globoid or racket-shaped particles in cytoplasm were observed under TEM in group B and C (yellow arrows). In addition, microcalcification surrounded by globoid human NPs within the seminiferous tubules increasingly occurred in affected tubules in group C (white arrows). **d** At four weeks, microcalcification surrounded by cellular debris within the seminiferous tubules displayed the characteristic features of testicular microlithiasis in group C only (Fig. 3d; red arrow)

In the testis of control rats, a normal cell population was found typically localized in the seminiferous tubules while no morphological changes were discovered (Fig. 3a). In the testis at 2 weeks, consistent with the histological findings, many globoid or racket-shaped particles in cytoplasm were observed under TEM in group C (Fig. 3b and c). In addition, microcalcification surrounded by globoid human NPs within the seminiferous tubules increasingly occurred in affected tubules in group C (Fig. 3c), which suggests that the formation of microcalcification is likely linked with NP exposure. At 4 weeks, microcalcification surrounded by cellular debris within the seminiferous tubules displayed characteristic features of testicular microlithiasis in group C only (Fig. 3d).

**Fig. 4:**
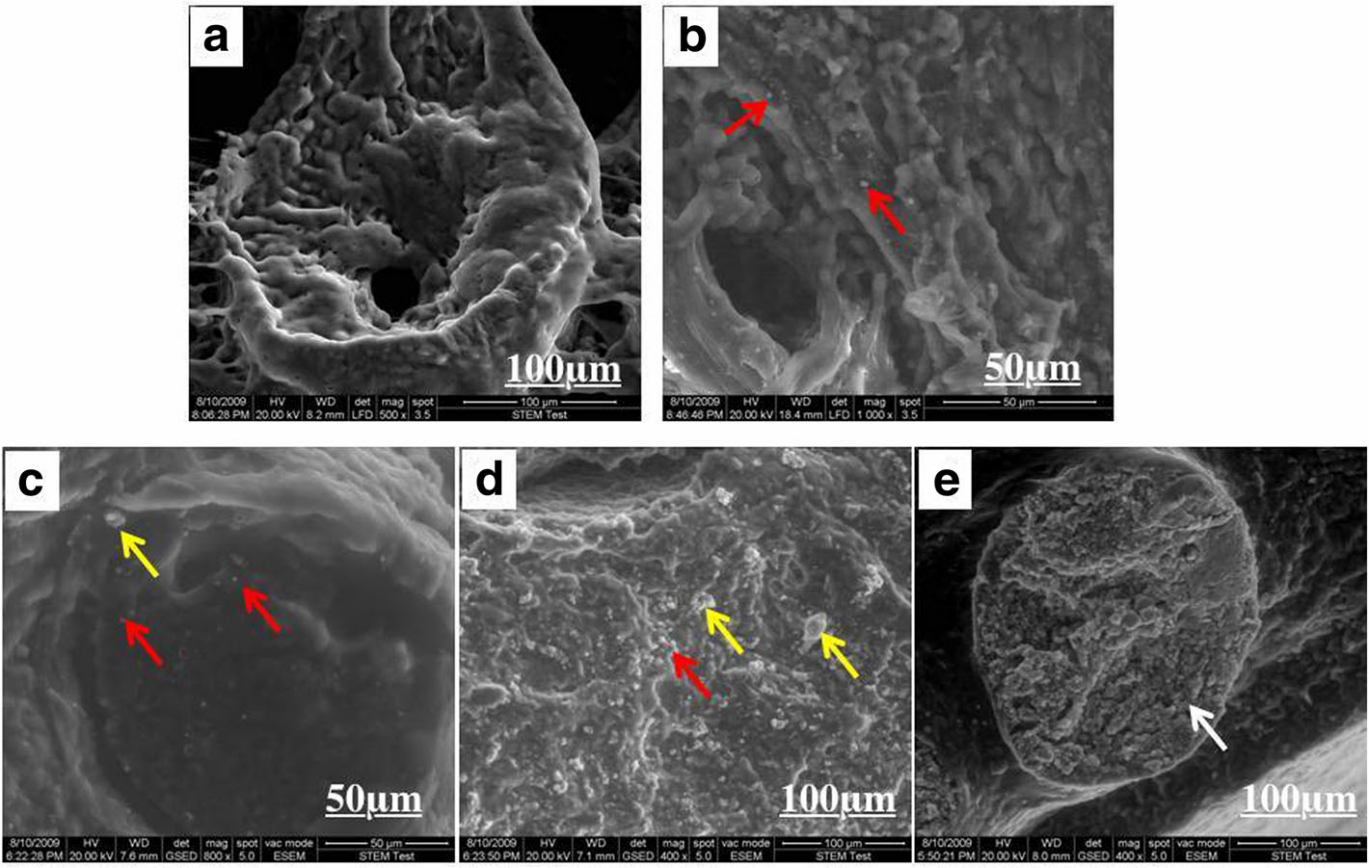
Testis ultrastructural observations by SEM. **a** An intricate three-dimensional network and each seminiferous tubule with large fenestrae and well spaced cells were observed in the testis of control mice. **b**-**d** At 2 weeks, distinct globoid particles were observed between the interstitial tissue and the seminiferous tubules in the testis after 2 week in group C (Fig. 4b and c; red arrows). In addition, the seminiferous tubules showed a compact, fibrous appearance with absence of fenestrae, but with a larger number of cellular debris, and microcalcification surrounded by globoid NPs within the seminiferous tubules were obviously observed in affected tubules in group C (Fig. 4c-d; yellow arrow). **e** At 4 weeks, the epithelium height diminished and no spermatozoa in the lumen could be witnessed except microcalcification filled in the seminiferous tubule only in group C (Fig. 4e; white arrows)

The SEM examination showed the interstitial tissue of control rats with an intricate three-dimensional network and each seminiferous tubule with large fenestrae and well spaced cells (Fig. 4a). Distinct globoid particles were observed between the interstitial tissue and the seminiferous tubules in the testis after 2 weeks in group C (Fig. 4b and c). In addition, the seminiferous tubules showed a compact, fibrous appearance with absence of fenestrae, but with a larger number of cellular debris, and microcalcification surrounded by globoid NPs within the seminiferous tubules were obviously visble in affected tubules in group C (Fig. 4c-d). At 4 weeks, the epithelium height significantly diminished and no spermatozoa in the lumen could be witnessed except microcalcification filled in the seminiferous tubule only in group C (Fig. 4e).

## Discussion

The emergence of nanobacteria, which are nanometer-scale spherical particles capable of producing nucleate hydroxyapatite, has spurred a major controversy in the field of microbiology [6, 7]. The absence of a fairly accurately sequenced genome for nanobacteria has led to various hypotheses of the characteristic and origin of nanobacteria. In this study, our cultured NPs successfully caused testicular inflammation, which suggested that they might be living entities.

Human NPs have been found mainly excreted from urinary tract and may flow back into seminiferous tubules, colonize there and cause testis infection [10, 12, 13]. To date, NPs have been found in periodontal diseases, urolithiasis, aortic valve calcification, human arthritic synovial fluid, and demonstrated to participate in the clinical pathological process of those diseases [10, 12, 13]. In a previous study, NPs were successfully cultured from 58.8% of semen samples of patients with TM [10]. Therefore, experimental studies on animal models focused on the uncovering of new connections between NPs and the etiopathogenesis of TM were needed.

To exclude the possibility of false positives of human NPs cultured from semen samples, three efforts were made in our study. Firstly, our experimental operations and testing instruments were conducted under strict aseptic conditions. Secondly, samples were cultured without fetal bovine serum, which usually contains inhibitors on apatite crystal formation, such as osteopontin, osteocalcin, and fetuin. Thirdly, the supernatants of human NPs cultures were filtered with a 0.22-μm minipore filter, which could effectively remove the common bacteria, mycoplasma, and fungi. In this study, human NPs were successfully cultured with serum-free RPMI-1640 from semen samples of patients with TM, which exhibited as spheroids with black coats and crystals around the bacterial body under TEM. All these results obtained from our experiments indicated that human NPs did reside in the testicular architecture of TM patients.

Furthermore, we injected the human NPs suspension into the male Sprague-Dawley rat proximal right ductus deferens, and pathological changes of the rats resulting from NP infection in the testis tissue were observed. Our investigation demonstrated that human NPs administration caused significant histopathological alterations in testis, manifested as leukocytic infiltration in the innermost layer of sperm cells at 2 weeks, structurally impaired sperm cells of the innermost layer and a zone of concentrically layered collagen fibers around the NP body formed in the lumen of the seminiferous tubules at 2 weeks, and a well-recognized form of intratesticular calcification surrounded by cellular debris within the seminiferous tubules at 4 weeks. However, no significant pathological changes were observed in the controls. Although significant leukocytic infiltration changes in the innermost layer of sperm cells were also observed in the groups receiving *E. coli* at 2 weeks, no intratesticular calcification surrounded by cellular debris within the seminiferous tubules was observed after 4 weeks. Therefore, a model of TM phenotype was successfully reproduced in this study. Our results also confirmed that human NPs were the known agents of emerging infectious diseases and producing apatite, which agrees well with other reports [14, 15]. A possible calcification mechanism might be NP colonization in seminiferous tubules inducing an immune response, promoting inflammation development and further calcification.

Ultrasound (US) is an effective approach for monitoring formation of calcification, which can be seen as tiny bright echoes without acoustic shadow scattered throughout the testicular parenchyma. Unfortunately, we failed to find a suitable ultrasonic probe for rats. Instead we did testis ultrastructural study at different points. TEM and SEM illustrations showed that the pathological alterations and calcification in seminiferous tubules were not homogenous. Many globoid or racket-shaped particles in cytoplasm were only observed in the NP-instilled rats and were not present in any of the controls under TEM. Moreover, the fibrils and mineral deposits developed in seminiferous tubules had different sizes and morphology under SEM. These illustrations further proved that human NPs did participate in the formation of testicular microlithiasis.

The present study was a descriptive rather than mechanistic study. In our early experimental design, we intended to explore the formation mechanism of testicular microlithiasis by NPs, and thus chose *E. coli* as a control to uncover the differences in histopathological alterations. Unfortunately, we failed to observe any significant differences in terms of histological features. Further investigations on the differences of the leucocyte population by means of immunohistochemistry (IHC) between them may better define the character of NPs and their possible mechanistic role in TM pathogenesis. Therefore, more effort addressing this question is needed in our future work.

## Conclusions

The results obtained from our study suggest a potential pathogenic effect of systemic inoculation of human-derived NPs to stimulate calcification within the seminiferous tubules.

## Abbreviations

NPs: nanoparticles
SEM: scanning electron microscopy
TEM: transmission electron microscopy
TM: testicular microlithiasis
